# Ferroptosis: A New Road towards Cancer Management

**DOI:** 10.3390/molecules27072129

**Published:** 2022-03-25

**Authors:** Iqra Bano, Pavel Horky, Syed Qamar Abbas, Muhammad Majid, Akram Hafiz Muhammad Bilal, Fawad Ali, Tapan Behl, Syed Shams ul Hassan, Simona Bungau

**Affiliations:** 1 Faculty of Bio-Sciences, SBBUVAS, Sakrand 67210, Pakistan; 2 Department of Animal Nutrition and Forage Production, Mendel University in Brno, 61300 Brno, Czech Republic; pavel.horky@mendelu.cz; 3 Department of Pharmacy, Sarhad University of Science and Technology, Peshawar 25000, Pakistan; qamar0613@yahoo.com; 4 Department of Pharmacy, Capital University of Science and Technology, Islamabad 44000, Pakistan; majidpharma808@gmail.com; 5 Key Laboratory of Advanced Drug Preparation Technologies, Ministry of Education of China, School of Pharmaceutical Sciences, Zhengzhou University, Zhengzhou 450001, China; bilalakram082@outlook.com; 6 Department of Pharmacy, Kohat University of Science and Technology, Kohat 26000, Pakistan; fawad.alee@gmail.com; 7 Department of Pharmacology, Chitkara College of Pharmacy, Chitkara University, Rajpura 140401, India; tapanbehl31@gmail.com; 8 Shanghai Key Laboratory for Molecular Engineering of Chiral Drugs, School of Pharmacy, Shanghai Jiao Tong University, Shanghai 200240, China; 9 Department of Natural Product Chemistry, School of Pharmacy, Shanghai Jiao Tong University, Shanghai 200240, China; 10 Department of Pharmacy, Faculty of Medicine and Pharmacy, University of Oradea, 410028 Oradea, Romania

**Keywords:** cancer, ferroptosis, molecular mechanisms, iron, tumor

## Abstract

Ferroptosis is a recently described programmed cell death mechanism that is characterized by the buildup of iron (Fe)-dependent lipid peroxides in cells and is morphologically, biochemically, and genetically distinct from other forms of cell death, having emerged to play an important role in cancer biology. Ferroptosis has significant importance during cancer treatment because of the combination of factors, including suppression of the glutathione peroxidase 4 (Gpx4), cysteine deficiency, and arachidonoyl (AA) peroxidation, which cause cells to undergo ferroptosis. However, the physiological significance of ferroptosis throughout development is still not fully understood. This current review is focused on the factors and molecular mechanisms with the diagrammatic illustrations of ferroptosis that have a role in the initiation and sensitivity of ferroptosis in various malignancies. This knowledge will open a new road for research in oncology and cancer management.

## 1. Introduction

Cell death is considered the heart of most disease processes, and it is also a critical component in the control of normal tissues [1]. It signifies the end of a cell’s existence. For a long time, scientists believed that there were two types of cell death, necrosis, and apoptosis or regulated cell death (RCD) [2]. Previously, RCD was thought to be identical with caspase-dependent apoptosis, but the discovery of various forms of nonapoptotic RCD showed that this was not always the case [3]. The receptor-interacting protein kinase-1 (RIPK-1)-dependent necroptosis, apoptosis-inducing factor-1 (AIF-1)-dependent parthanatos, and iron (Fe)-dependent ferroptosis are all kinds of cell deaths [4]. Dixon suggested ferroptosis as a new mode of cell death for the first time in 2012 [5]. Ferroptosis, in contrast to autophagy and apoptosis, is a cell death that is primarily characterized by cytological alterations, such as reduced or disappeared mitochondrial cristae, a torn outer mitochondrial barrier, and a compacted mitochondrial membrane [6]. These cell abnormalities are caused by the loss of selective permeability of the plasma membrane, which occurs because of severe lipid peroxidation reactions [7]. Recently, ferroptosis is being more recognized as an adaptive characteristic that helps the body destroy cancerous cells [8]. It plays a critical role in the prevention of tumor formation by eliminating cells that are lacking in essential nutrients and water and that have been damaged by infection or environmental stress [2]. The traditional oxidative stress (OS) route was a significant causal component in the development of ferroptosis, according to research. Although cancer cells are subjected to the constant OS and have achieved an excellent equilibrium among thiols and catalytic Fe, ferroptosis does not occur often throughout cancer growth [7]. The cell organelles and many signaling pathways have been involved in the control of ferroptosis [9]. Small compounds have also been discovered that can trigger ferroptosis in a broad spectrum of cancer cells. Scientific and medical researchers are eager to explore the prospect of cancer therapeutics based on genetic or pharmaceutical involvement with ferroptotic cell death [7]. Ferroptosis engages in the inception and development of a wide range of projects. For cancer treatment, ferroptosis sensitivity in tumors from diverse tissues will also be beneficial [10]. Several aspects of ferroptosis initiation and control are discussed in this review, including the small molecules and signaling pathways involved, and the cell organelles that play a role in ferroptosis regulation. In addition, we discuss the use of ferroptosis in overcoming drug resistance in cancer cells from many angles

## 2. Mechanism of Ferroptosis

### 2.1. Ferroptosis Due to Suppression of System Xc^−^

**Figure 1:**
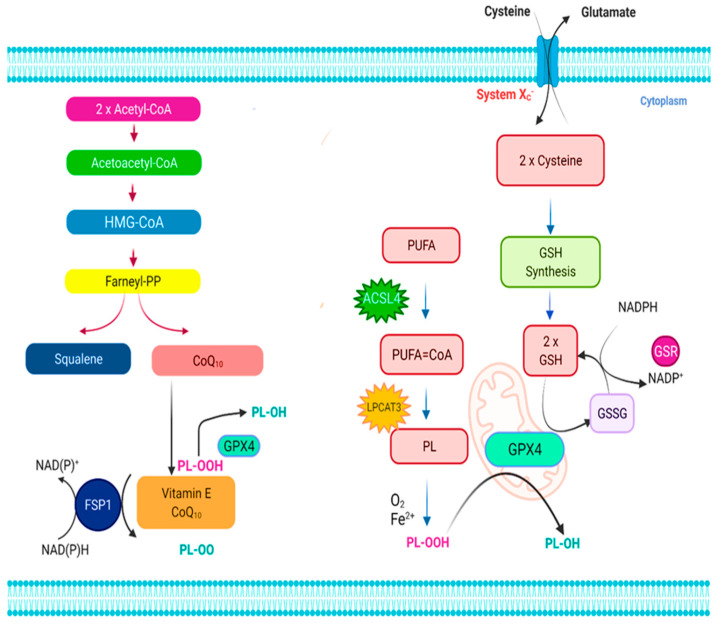
The ferroptosis signaling pathway. Ferroptosis is regulated by two major regulatory mechanisms, namely the cystine/glutathione (GSH)/glutathione peroxidase 4 (Gpx4) axis and the NAD(P)H/ferroptosis suppressor protein 1 (FSP1)/ubiquinone (CoQ10) axis. The cystine/glutathione (GSH)/glutathione peroxidase 4 (Gpx4) axis regulates ferroptosis suppression through the cystine/GSH/Gpx4 nexus is accomplished by the main stages of cystine absorption and decrease, respectively, GSH production, and Gpx4 activation, among others, and the conversion of oxidized phospholipids (PLs) into their homologous alcohols (PL-OH) by the enzyme Gpx4, which uses GSH as a substrate. It has been shown that the enzymes acyl-CoA synthetase long-chain family member 4 (ACSL4) as well as lysophosphatidylcholine acyltransferase 3 (LPCAT3) are involved in integrating polyunsaturated fatty acids (PUFAs) within cellular membranes, making them susceptible to ferroptosis activation. As a result, the oxidation of lipid bilayers may proceed enzymatically and/or nonenzymatically by auto-oxidation, depending on the situation. The antiferroptotic activity of FSP1 in the NAD(P)H/FSP1/CoQ10 system is attributed to its oxidoreductase activity, which is activated by converting extramitochondrial CoQ10 to ubiquinol using NAD(P)H/H+. To avoid lipid peroxidation, ubiquinol either reduces lipid radicals (PL-OO) directly or indirectly via the use of vitamin E (-tocopherol) must be present. Apart from that, the mevalonate pathway produces precursors for CoQ10 and squalene, the latter of which is beneficial in the prevention of ferroptosis because of its antioxidant action.

A heteromeric amino acid transporter, system Xc^−^, is expressed in the plasma membrane of many types of cells and is the target of type I ferroptosis activators (erastin, sulfasalazine, and sorafenib) [11]. The system Xc^−^ mediates the import and export of cystine and glutamate from the cell. [12]. Glutamate and cysteine are exchanged in a 1:1 ratio to regulate the redox status of cancer cells through the membrane-anchored system Xc^−^. To avoid entering a peroxidation state, cancer cells use system Xc^−^ to regulate their redox status and metabolism, which ultimately aids tumor growth [13]. A reduced form of cystine, cystine dihydrochloride (Cysteine), serves as a precursor for manufacturing glutathione (GSH) [14]. The Gpx4 catalysis lipid peroxides by exerting its phospholipid peroxidase activity in the presence of GSH, which serves as a crucial cofactor [15]. The suppression of system Xc^−^ by small molecules results in GSH depletion, which leads Gpx4 to be inactivated, resulting in the buildup of fatal lipid peroxides and the onset of ferroptosis [16]. However, it should be noted that suppression of the gamma-glutamyl-cysteine (g-GCS), e.g., via buthionine sulfoximine agents also suffices to cause ferroptosis in certain cases (Figure 1) [17]. 

### 2.2. Ferroptosis Involving Gpx4

Gpx4 has been shown to reduce mitochondrial apoptosis in preliminary studies. This suggests that whereas Gpx4 is primarily responsible for controlling ferroptosis, its absence in some cell types may also result in other types of cell death. Ferroptosis and other cell death processes are yet unknown. It is unclear whether the subcellular location of lipid peroxidation is important in inducing ferroptosis or if ferroptotic cell death executors are still unidentified [11]. Researchers have revealed that ferroptosis is caused by direct suppression of Gpx4 by either decreasing its activity or encouraging its destruction [7]. Irreversibly, the traditional ferroptotic activator (1S, 3R)-RSL3 may reduce the enzymatic activity of the Gpx4 enzyme by covalently engaging its active site selenocysteine [18]. A novel ferroptosis inducer known as FIN56 is involved in reducing the amount of Gpx4 present [19]. While the precise process is still unknown, it may have something to do with the catalase activity for acetyl-CoA carboxylase [20]. Genetic suppression of Gpx4 via siRNA caused a buildup of lipid reactive oxygen species (ROS), causing ferroptotic death, as shown by these results. Generically, the protein Gpx4 acts as a major regulator of ferroptosis-triggering pathways [21]. Since cells experiencing ferroptosis die because of a Fe-dependent buildup of ROS, combined Fe metabolism plus lipid peroxidation are two important methods employed in the pathogenesis of ferroptosis [22]. Gpx4-regulated ferroptosis is also highly sensitive to diffuse large B cell lymphomas and renal cell carcinomas, according to sensitivity profiling of 177 cancer cell lines [23].

### 2.3. Ferroptosis Involving Mitochondrial Voltage-Dependent Anions Channels

The mitochondrial voltage-dependent anions channels (VDACs) proteins are beta-barrel porins that span the outermost mitochondrial membrane and are responsible for permeability. As the most prevalent outer mitochondrial membrane protein, VDAC1 is the first of three isoforms, VDAC2 is the second, and VDAC3 is the third [24]. Glycolysis is the principal energy source for malignant cells if there is enough oxygen available to the cells to do so. Statistically speaking, this is called the Warburg effect [25]. After tubulin blocks VDAC and closes it, the flow of respiratory substrates into the mitochondria is restricted. In this way, the Warburg effect can be activated, resulting in cancer cell aerobic glycolysis. Erastin’s ability to control VDAC opening will have a substantial impact on mitochondrial metabolism, according to this study [26]. An increase in oxidative phosphorylation will lead to an increase in ROS generation and a reversal of the Warburg phenotype, which is associated with aerobic glycolysis. Increased mitochondrial ROS and oxidative stress will result, and ferroptosis will occur [27].

### 2.4. Some Other Pathways Involving Ferroptosis

Genetic screenings conducted throughout the entire genome later identified an enzyme called acyl-CoA synthetase long-chain family member 4 (ACSL4), which was used to measure the susceptibility of cells to ferroptosis after being discovered by accident [28]. Activation of long-chain polyunsaturated fatty acids (PUFAs) is thought to have a role in ferroptosis because these PUFAs may enhance the likelihood of the formation of lipid peroxidation when they are included in phospholipids [29]. Ferroptosis is characterized by lipid peroxidation and the rupture of cellular membranes. However, it is still debated whether this process is mediated by specific lipoxygenase or whether it is caused either by Fe-dependent, radical-mediated Fenton reaction, as well as autoxidation of lipid bilayers [18]. Lipid peroxidation inhibitors or efforts to reduce the amount of PUFAs on the membrane surface are the most effective ways to avoid ferroptosis. Similarly, Fe chelation has been shown to protect against OS and ferroptotic death of cells frequently [21]. The specific involvement of Fe in ferroptosis is something that has to be investigated further [30]. In addition to autophagic destruction of the Fe-storing protein ferritin, heme oxygenase-1-mediated heme degradation, and the reaction to Fe deprivation, there are other putative intracellular contributors to labile redox-active Fe [31]. In the search for previously unidentified ferroptosis resistance mechanisms, two independent research groups revealed ferroptosis suppressor protein-1 (FSP-1) previously known as ‘apoptosis-inducing factor mitochondria associated 2’ (AIFM2) as a novel ferroptosis regulator [32]. FSP-1 was discovered to be a very effective antiferroptotic enzyme, and overexpression of this enzyme resulted in full rescue from ferroptosis produced by chromosomal deletion or pharmacological suppression of Gpx4 (a ferroptotic enzyme) [33]. Because of its oxidoreductase activity, FSP-1 has an antiferroptotic effect on extramitochondrial ubiquinone (CoQ10), which it converts to ubiquinol by reducing it with NAD(P)H/H+ [34]. Other mechanisms, including sulfur transfer pathways, may play a role in the incidence of ferroptosis. When exposed to OS, methionine may be transformed into cystine through the sulfur transfer route, and then GSH can be produced to further enhance the antioxidant properties of the amino acid [35]. The P62-Kelch-like ECH-associated protein 1- nuclear factor erythroid 2-related factor 2 (p62-Keap1-Nrf2), Autophagy-related-5, autophagy-related-7, and nuclear receptor coactivator 4 autophagy-related-5 pathways (ATG5-ATG7-NCOA4), and glutamine metabolic pathways, in addition, can efficiently govern the generation of cellular Fe ions and ROS, as well as play a regulatory role in ferroptosis [36]. Several signal transductions channels and events in cells are involved in ferroptosis’s complex molecular process [21]. In this way, ferroptosis is regulated and monitored by several ferroptosis-related proteins that are involved in cystine absorption, Fe metabolism, fatty acid metabolism, OS, and mitochondrial biogenesis (Table 1).

### 2.5. Role of Mitochondria in Ferroptosis

Mitochondria are the energy-producing organelles of the cell, and they have long been thought to be intimately associated with the process of programmed cell death [46]. Despite this, the involvement of mitochondria in ferroptosis continues to be a source of intense debate. It was Dixon’s research that showed that ferroptosis might occur in cells without a functioning mitochondrial electron transport chain (ETC), as seen in HT1080 cells [47]. Gao et al. demonstrated that reduction of the mitochondria by parkin-mediated mitophagy significantly reduced the susceptibility of cells towards cysteine deprivation-induced ferroptosis, as previously noted in the literature [46]. Attenuation of cysteine deprivation-induced ferroptosis may be achieved by inhibiting the mitochondrial tricarboxylic acid (TCA) cycle or the ETC [48]. Although the mitochondria are involved in glutaminolysis and the TCA cycle, mitochondrial lipids seem to be key sources of lipid peroxides during ferroptosis [33]. They did, however, discover that mitochondria were only involved in cysteine deprivation-induced ferroptosis, and not in Gpx4 inhibition-induced ferroptosis, as previously thought [49]. In cysteine deficiency states, the mitochondria may work upstream on Gpx4 to enhance the depletion of GSH, which would explain why this occurs [29]. In cardiomyocytes, it was shown that mitochondria play critical roles in ferroptosis control [50]. During doxorubicin-induced cardiac ferroptosis, lipid peroxidation and non-haem Fe levels were raised primarily in the mitochondria, though not in the cytosol [47]. Furthermore, genes involved in mitochondrial fatty acid metabolism, including citrate synthase and acyl-CoA synthase family member 2 (ACSF2), are thought to be necessary for erastin-induced ferroptosis [30]. These data showed the mitochondria play an important role in the induction of ferroptosis. The discrepancies in data regarding the functions of mitochondria for ferroptosis may be related to the use of various methodologies for measuring cell death [49]. Because modulation of mitochondrial activity may influence the result, the cellular respiration assays will not be adequate for the investigation of the involvement of mitochondria in ferroptosis [6].

### 2.6. Role of Lysosome in Ferroptosis

Lysosomes are also involved in the induction of ferroptosis. According to the fluorescent ROS sensors, the lysosome is the most important cellular ROS reservoir in HT1080 cells that have been exposed to erastin or RSL3-induced ferroptosis [51]. Moreover, lysosome activity inhibitors may prevent both lysosomal ROS and a ferroptotic cell death-associated ROS burst, suggesting that they might treat both conditions [21]. The activity of lysosomes affects intracellular Fe supply through inhibiting intracellular trafficking of transferrin via autophagic degradation of ferritin, among other mechanisms [52]. Another work by Gao et al. has also established the relevance of the lysosome in ferroptosis. discovered that inhibiting lysosome cathepsin B, a cysteine proteinase, lowered the susceptibility of cells to erastin-induced ferroptosis in their experiments [46]. Among the human pancreatic ductal adenocarcinoma (PDAC) cell lines, signal transducer and activator of transcription 3 (STAT3) are implicated in ferroptosis through modulating the production of cathepsin B [53]. In recent research, ferroptosis was identified as an autophagic cell death, which further highlights the critical role played by the lysosome in ferroptosis, since the lysosome is the primary organelle responsible for the autophagic destruction of protein aggregates [6].

### 2.7. Role of Fe Metabolism in Ferroptosis

In the human body, Fe is a vital trace element. Human physiological functions may be disrupted by abnormalities in Fe distribution and content [54]. When it comes to bringing Fe into the body, ferroportin (Fpn) is a vital component that converts excess Fe^2+^ to Fe^3+^. Fpn is also the only recognized transporter which imports elemental Fe from cells, and appears as a crucial carrier in terms of ferrous acquisition and distribution between cell types [55]. One way by which Fpn is regulated is by its association with hepcidin, a polypeptide generated by the body in response to Fe storage and the processes of erythropoiesis and hypoxia as well as inflammation [56]. Hepcidin unites Fpn, which causes it to be taken inside and then broken down by the body. The discharge of Fpn from the surface of the cell accomplishes a homeostatic sequence in which the flow of Fe is coordinated with the amount of Fe the body needs [57]. Transferrin (TF) on the cell surface binds to Fe^3+^, which may be oxidized by ceruloplasmin to generate TF-Fe^3+^, which forms complexes via the protein complex TF receptor-1 (TFR-1) to enter the cell [58]. Thereafter, six transmembrane epithelial antigens of the prostate-3 (STEAP-3) reduce Fe^3+^ to Fe^2+^, which is stored in the labile iron pool (LIP) and ferritin, which is regulated via divalent metallic transporter-1 (DMT-1) and Zinc-Iron regulatory protein family 8/14 (ZIP8/14) [59]. The cellular Fe homeostasis is tightly regulated by this process of Fe recycling inside the cell [52]. The erastin-induced ferroptosis may be inhibited by silencing transferrin receptor (TFRC), the protein that encodes the TFR-1 gene [36]. Moreover, the erastin-induced ferroptosis may be sped up by adding Fe with heme oxygenase-1 (HO-1) [60]. Overexpression of heat shock protein beta-1 (HSPB1) dramatically inhibits ferroptosis by reducing intracellular Fe concentrations through suppressing TRF-1 expression [48]. Aside from that, ferritin comprises two different chains: ferritin light chain (FTL) as well as ferritin heavy chain-1 (FTH-1). Ferroptosis and NCOA4 degradation of mitochondrial ferritin (FTMT) have recently been demonstrated. Results indicated that hypoxic primary human macrophages reduced levels of free Fe and enhanced ferritin expression, particularly FTMT, for Fe storage. FTMT expression was shown to be regulated by the NCOA4, as a kind of cell death associated to Fe metabolism, FTMT, and FTH-1 [61]. Much recent research has shown that Fe homeostasis and ferroptosis work hand-in-hand for the treatment of tumors. To keep Fe levels in check, the iron regulatory proteins IRP1 and IRP2 play a critical role. Ferroptosis caused by erastin and RSL3 was dramatically reduced by depletion of IRP1. IRP2 showed a small effect on ferroptosis, but it had the potential to augment the promoting function of IRP1 [62]. Moreover, another study revealed that while decreasing the transcription of iron response element-binding protein-2 (IREB-2), the primary transcription factor of Fe metabolism may enhance the expression of FTL and FTH-1, hence inhibiting ferroptosis triggered by erastin [63].

## 3. Connections between Ferroptosis and Other Forms of Cell Death

Ferroptosis is characterized by increased mitochondria membrane density, the disappearance of mitochondrial cristae, and the burst of the mitochondrial outer membrane, reduction in the size of mitochondria which distinguishes it from apoptosis, necrosis, as well as autophagy in terms of morphological features [64]. Ferroptosis is distinct from apoptosis, necrosis, and autophagy in terms of morphological features. Furthermore, inhibitors of apoptosis, necrosis, or autophagy are unable to prevent ferroptosis from occurring. However, since the discovery of ferroptosis, researchers have continued to investigate the connection between ferroptosis and other types of cell death indefinitely [49]. According to recent research, ferroptosis shared a few characteristics with several other forms of cell death including decreased or vanished mitochondria cristae, cytological modifications as well as condensed mitochondrial membranes [47]. However, even though cells experiencing ferroptosis show mitochondrial impairment, ferroptotic cell death cannot be attributed to mitochondrial damage since the levels of ROS in mitochondria in the samples transfected with erastin remain constant in the treated cells [52]. Furthermore, ferroptosis may occur in cells that do not have a functioning mitochondrial ETC, which is the route via which ROS are produced in mitochondrial membranes as stated above [54].

### 3.1. Necrosis and Ferroptosis

Ferroptosis is different from necrosis and apoptosis, although a few studies have shown that ferroptosis may occur in conjunction with necroptosis in certain cases [65]. Unlike necrosis, ferroptosis results in mitochondrial shrinkage and an increase in mitochondrial membrane density, rather than chromatin condensation, degradation of plasma membrane integrity, or the emergence of double membrane-layered autophagic vacuoles [41]. Ferroptosis inhibitors are drugs that prevent ferroptosis from occurring. According to the literature, the OS was reduced by ferroptosis inhibitors (e.g., hemin, ferrostatin-1) as well as necroptosis inhibitors (e.g., deferoxamine, trolox) [65]. Hemin-induced cell death also raised the levels of ferroptosis molecular indicators (phospho-ERK1/2) as well as necroptosis biomarkers (RIP1 and RIP3) in mRNA. Although cell death caused by hemin seems necrotic under electron microscopy, there are no shrinking mitochondria to be seen, which is a hallmark of ferroptosis. Necrosis causes the deterioration of membrane stability as well as organelle disruption [49].

### 3.2. Ferroptosis and Oxytosis

Neuronal cells undergo oxytosis, a kind of oxidative cell death. The glutamate-mediated inhibition of the system which results in the depletion of GSH causes oxytosis to occur. In neural cells, oxytosis is a kind of cell damage death that occurs [66]. Oxytosis is triggered by the glutamate-mediated blocking of system Xc^−^, which results in the depletion of GSH levels. The depletion of GSH impairs the antioxidant defense of the cells and causes the buildup of ROS in the body [51]. In contrast to ferroptosis, glutamate-induced oxytosis seems to be caused by the same mechanism as erastin-mediated ferroptosis [48]. Ferroptosis, on the other hand, is distinguished from oxytosis by the presence of the BH3 interacting domain death agonist protein (BID), which maintains mitochondrial integrity and function [67]. Both oxytosis, as well as ferroptosis, are prevented by BID knockout and BID inhibitors. It is noteworthy that the ferroptosis-specific inhibitors ferrostatin-1 and liproxstatin-1 both restore glutamate-induced oxytosis and maintain mitochondrial integrity in the presence of glutamate [15]. Among the distinctions between ferroptosis as well as oxytosis is the fact that ferroptosis is unable to transactivate BID and also that oxytosis does not exhibit the critical apoptosis-inducing factor (AIF) translocation with ferroptosis. Evidence of a relationship between these two forms of cell death is required to be established with more certainty [68].

### 3.3. Autophagy and Ferroptosis

When autophagy takes place in the cell, proteins and organelles are collected in autophagosomes and then sent to the lysosomal compartment where they are degraded [10]. The inhibition of autophagy-mediated via autophagy-related protein-13 (ATG-13), as well as autophagy-related-3 protein (ATG-3) knockdown, significantly decreased cysteine deprivation-driven ferroptosis in a dose-dependent manner [51]. Autophagy may have a role in the promotion of ROS generation and the consequent buildup of lipid peroxides in the body [3]. Researchers also claim that the ferritinophagy, which is mediated by the nuclear receptor coactivator-4 (NCOA-4), will degrade ferritin and liberate Fe, allowing for the development of ferroptosis [6]. A reduction in ferritinophagy and an abundant supply of free Fe result from NCOA-4 knockdown, which prevents the buildup of ROS and the activation of ferroptosis. However, there is currently a paucity of data to establish that ferroptosis directly results from autophagy in the human body [9]. During times of low intracellular Fe, NCOA-4 interacts with the FTH-1 in autophagosomes, causing the autophagosomes to be transported to lysosomes for ferritin breakdown [69]. Fe was increased in senescent cells because of defective ferritinophagy and suppression of ferroptosis. Autophagy, also known as ferritinophagy, increased ferroptosis in fibroblasts and tumor cells by increasing the quantity of Fe in the cells via the destruction of ferritin [70]. In the presence of liver fibrosis, erastin-induced ferroptosis was associated with the activation of ferritinophagy. According to these observations, ferroptosis was associated with the activation of ferritinophagy, or ferritinophagy was associated with the promotion of ferroptosis [38]. However, research done in 2016 discovered that inhibiting autophagy/ferritinophagy with a particular inhibitor and knocking out NCOA-4 were effective in preventing ferroptosis in mouse embryonic fibroblasts as well HT1080 cells, respectively. An autophagic mechanism known as ferroptosis was described in this study as a prerequisite for the onset of autophagy [71].

## 4. Ferroptosis and Cancer

In tumor formation or therapy, several kinds of controlled cell death, such as autophagy, apoptosis, and necrosis, play critical roles [10]. Ferroptosis has been discovered in a variety of cancers, including breast cancer, renal cell carcinoma (RCC), lung cancer (LC), pancreatic cancer (PC), diffuse large B-cell lymphoma (DLBCL), head and neck squamous cell carcinomas (HNSCC), and hepatocellular carcinoma, (HCC) in recent years [72]. Ongoing research is investigating the stimulation of cell death as a cancer treatment method. BJeLR cell-derived xenograft mice models with prostaglandin-endoperoxide synthase-2 (PTGS-2) upregulation, which is a particular marker for RSL3 and erastin-induced ferroptosis within tumors, were shown to have decreased tumor development when treated with RSL3, which is a ferroptosis-inducing agent [42]. Furthermore, Gpx4 knockdown results in ferroptosis in renal cell carcinoma cells, which is accompanied by an increase in lipid ROS [38]. Erastin can be used as an anticancer treatment in a variety of cancers when combined with other drugs, such as cisplatin, temozolomide, cytarabine/ara-C, and adriamycin, among others [73]. Recent research discovered the first evidence of a relationship between ferroptosis and the radiation-induced bystander effect (RIBE). Because of the findings of this research, it is hypothesized that irradiated tumor-cell released microparticles (RT-MPs) are indeed the primary mediator of RIBE, which results in widespread antitumor effects via ferroptosis. These results suggest that ferroptosis may have a role in the advancement of cancer or the control of cancer growth [74]. In this paper, we discuss the significance of ferroptosis in cancer, specifically how it interacts with the tumor suppressor genes p53, malignant cells noncoding RNA (ncRNA), and the tumor microenvironment.

### 4.1. Ferroptosis and Tumor Microenvironment

There are a variety of noncancer cells in the tumor microenvironment (TME) that play an important role in the development and progression of cancer, as well as in the disease’s treatment [75]. Cancer cells may theoretically be made more resistant to immune surveillance by altering their cytogenetics and epigenetics, which might lead to an increase in neoplasm growth, progression, and metastasis [76]. Ferroptosis may be linked to cancer formation and suppression, according to new research. However, it is unclear what exactly ferroptosis does to the TME [65]. New and effective anticancer methods might be developed by studying the interplay among ferroptosis and the TME. As hypoxia is a well-known feature of the TME, it plays a role in carcinogenesis and treatment resistance [77]. The hypoxia-inducible factor (HIF) transcription factor family controls angiogenesis during cancer, which is triggered by hypoxia [78]. Interestingly, recent research discovered that the HIF pathway is that promotes the onset of ferroptosis in clear-cell carcinoma (CCC) [44]. As the hepatocyte growth factor-2 (HGF) regulates the production of hypoxia-inducible protein-2 (HIG-2) to increase lipid peroxidation by selectively enriching PUFA-lipids. The hypoxia-inducible factor-1 (HIF-1) depletion may also reduce the susceptibility of CCC cells to ferroptosis, showing that tumor cells may activate HIF-1 to imitate the hypoxia response in vivo [79]. The promoter region of several Fe metabolism genes such as FTH, transferrin receptor-1 (TFR1), as well as solute carrier family 11 member-A2 (SLC11A2) encoding divalent metal transporter-1 (DMT1), is controlled by hypoxia-responsive elements, which are found throughout the genome [9]. Hypoxia may aid cancerous mesothelioma cells in their defense towards ferroptosis by overexpressing the production of carbonic anhydrase-9 (CA9) [2]. Overexpression of CA9 in multiple myeloma (MM) cells under hypoxia lead to reduced Fe absorption and high Fe storage, which suppresses Fe-dependent OS and eventually results in the prevention of ferroptosis [6].

### 4.2. Ferroptosis and Tumor Suppressor p53

The tumor suppressor gene p53, sometimes known as the “defender of the genome”, is essential in the prevention and treatment of cancer [80]. It is thought to serve an antitumor role by causing cell cycle progression, apoptosis, and senescence in precancerous cells; however, it is also thought to control other processes including stem cellular functions, metabolism, propagation, and migration of cancerous cells [81]. Many studies have recently revealed that the tumor suppressor p53 may control genes associated with ferroptosis to prevent tumor formation [82]. The p53^3KR^ mouse (an animal that has lysine to arginine alterations at the p53 acetylation sites) does not spontaneously develop tumors, unlike the wild-type mouse [83]. Despite the absence of p53-dependent apoptosis, senescence, and cell cycle arrest, p53^3KR^ cells still can modulate SLC7A-11 expression [84]. Additional studies have shown that p53 targets SLC7A-11, spermidine/spermine N1-acetyltransferase 1 (SAT1), PTGS2, and glutaminase-2 (GLS2) to promote ferroptosis. For example, SAT1 leads to activation of arachidonate lipoxygenase 15-LOXs, which oxidize PUFAs and contribute to OS [85]. The fact that p53 may have an indirect influence on ferroptosis rather than directly aiding it is noteworthy [81]. This is because it may regulate the genes that it targets indirectly. Under some circumstances, p53 may reduce the vulnerability to ferroptosis [85]. Colorectal cancer (CRC) cells expressing the wild-type p53 gene are resistant against erastin-induced ferroptosis, whereas dipeptidyl peptidase-4 (DPP4), a lipid metabolism regulator also known as CD26, is responsible for this resistance [86]. This interaction results in the formation of an intermediate between DPP4 and the nicotinamide adenine dinucleotide diphosphate oxidase-1 (NADPH Oxidase/NOX-1), that also accelerates the DPP4-dependent, plasma membrane-associated fatty acid peroxidation and, as a result, induces lipid peroxidation deposition and ferroptosis [87]. Furthermore, the p53 inhibition may limit the nuclear aggregation of DPP4 in CRC cells, while p53 overexpression results in DPP4 being concentrated in the nuclei and not binding to NOX1 [88]. Other studies imply that the p53-p21 transcriptional pathways may be involved in the regulation of ferroptosis in tumor cells that had lower ROS production and slower intracellular GSH depletion than normal cells [89]. The p53 gene, as previously documented, serves conflicting functions of “brake” and “accelerator” in the control of ferroptosis in a variety of tumor cell types [90]. In this approach, p53 may influence ferroptosis susceptibility in a cell type-specific manner. However, ferroptosis may have physiological importance in the fact of evolution since it is a kind of RCD [81]. By enhancing the susceptibility of cells to ferroptosis, it may aid in the removal of aberrant cells or prevent tumor development. However, p53 also plays a critical role in ensuring that healthy cells can withstand a variety of stressors [44]. Certain tumor cells can avoid ferroptosis by maintaining the normal activity of the p53-p21 pathway in the face of increased ROS [10]. There is still much to learn about p53’s involvement in ferroptosis, but new research approaches for targeted tumor cell clearance and the conquering of cancer medication resistance will surely emerge as a result of this investigation [36].

## 5. Ferroptosis Inhibition in Different Forms of Cancer

**Figure 2:**
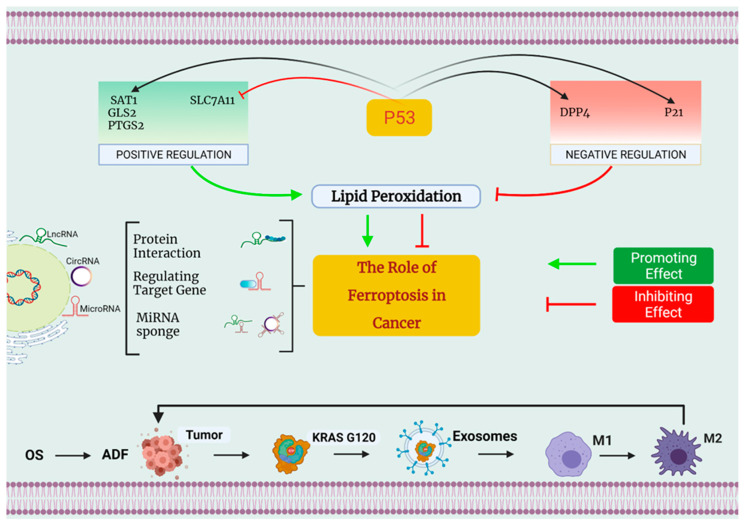
The involvement of ferroptosis in the development of cancer. First and foremost, p53 can control ferroptosis both favorably and negatively. On the one hand, p53 may promote ferroptosis by decreasing the expression of SLC7A11 while simultaneously inhibiting the regulation of SAT1, GLS2, and PTGS2. On the other hand, p53 can inhibit ferroptosis by enhanced expression of SAT1, GLS2, as well as PTGS2. On the other side, p53 can reduce ferroptosis by inhibiting DPP4 action and increasing the production of the protein p21. Second, small nucleolar RNA (snRNA) is a new regulator of ferroptosis control, and this includes long noncoding RNA (lncRNA), microRNA, and circRNA. Differential mechanisms that regulate ferroptosis may be classified into three types: some suggest that LINC00336 and circ-TTBK2 might act as competitor endogenous RNAs (ceRNAs), binding to miRNAs and regulating miRNA production, hence altering the ferroptosis process. The microRNAs MiR-214 and MiR-137 may influence the formation of genes implicated in ferroptosis. lncRNAs p53RRA enhance ferroptosis through interacting with ferroptosis-related proteins, according to research. Third, under conditions of OS, autophagy-dependent ferroptosis (ADF) promotes pro-tumorigenic M2 macrophage polarization and the production of tumor-associated macrophages (TAMs), both of which are necessary for cancer development.

Ferroptosis has indeed been linked to several disorders, including Parkinson’s syndrome and pancreatic cancer, among others [46]. The initiation or suppression of ferroptosis interferes with the progression of the illness [33]. Following the discovery that tumor cell sensitivity to ferroptosis is closely related to a variety of biological processes, including amino acid and Fe metabolism and PUFA and GSH metabolism, it has been hypothesized that interventions that target those biochemical mechanisms may regulate cancer cell sensitivity to ferroptosis [9]. According to emerging data, ferroptosis is blocked in malignancies such as HCC, breast cancers, pancreatic cancer, lung cancer, gastric cancer, cervical melanoma, and various cancers other forms of cancers [91]. It has been shown that ferroptosis suppression in cancer is connected with the activation of the system Xc^−^ importer, elevations in GSH metabolism and Gpx4 action, as well as suppression of OS and Fe metabolism (Figure 2) [92].

### 5.1. Breast Carcinoma

The solute carrier family 7 member 11 (SLC7A11) is a part of the cysteine-glutamate transporter which acts as a major component in tumorogenesis. In a similar way to SLC7A11, the MUC1-C (transmembrane oncoprotein) is connected to GSH production and causes redox stability in triple-negative breast cancer cell lines. Studies have revealed that MUC1-C interacts with CD44v (a variation of CD44), increasing the strength of SLC7A11 upon that cell membrane and resulting in an increase in GSH production and ferroptosis inhibitory activity [74]. Following treatment with Gpx inhibitors, the transcription of the pentaspanin protein (prominin-2), which has been involved in the control of lipid dynamics, increases in adherent cells such as breast cancer cells [93]. Because of the creation of ferritin-containing vesicles bodies and exosomes that induce Fe export, prominin-2 helps to prevent excessive buildup of free Fe in cells and the development of ferroptosis. Moreover, the ferroptosis is also increased by the suppression of multivesicular bodies, which suggests that prominin-2-induced exosome production may serve as a protective strategy against ferroptosis [94].

### 5.2. Pancreatic Carcinoma

It has been discovered that SLC7A11 is upregulated in pancreatic ductal adenocarcinomas (PDACs), which enhances cystine absorption and GSH production, hence promoting the development and existence of cancer cells by suppressing ferroptosis [95]. The heat shock protein-5 (HSPA5) is a transcription factor that negatively controls ferroptosis in human PDACs cells. Silencing of HSPA5 increases erastin-induced apoptosis in cells via the ferroptosis-dependent method [96]. The activating transcription factor-4 (ATF4) stimulates the production of HSPA5, which then in response interacts with Gpx4, resulting in the suppression of ferroptosis [88]. It is important to note that this ATF4-HSPA5-Gpx4 pathway, which mediates ferroptosis, also reduces the anticancer action of gemcitabine drugs [97].

### 5.3. Lung Carcinoma

According to emerging data, the BRCA1-associated protein-1 (BAP-1) reduces cystine absorption and GSH production by the SLC7A11 protein complex [98]. It becomes more difficult for cells to withstand the tremendous levels of accumulated peroxides because of GSH depletion, which eventually leads to the development of ferroptosis [99]. BAP-1 overexpression has been shown to decrease tumor cell proliferation both in vivo and in vitro. Because of this, BAP1 may act as a natural tumor suppressor gene, inhibiting the development of tumors [98]. The BAP1 gene is commonly deleted or altered in human cancer, including lung cancer, breast cancer, as well as renal cancer. In cancer cells, BAP1 inactivation leads to the overexpression of SLC7A11, the suppression of ferroptosis, and the formation of tumors [100]. High amounts of the SLC7A11 and SLC3A2 enzymes have been seen in certain lung cancer cells. When comparing ADCs and squamous cell carcinomas (SCCs) to normal lung tissues, SLC7A11 is considerably overexpressed [96]. According to a Kaplan–Meier (KM) analysis, increased SLC7A11 expression is related to a decreased 5-year survival rate in cancer patients [101]. It is thought that overexpressing SLC7A11 leads to a considerable reduction in the overall ROS level, therefore increasing the antioxidant capacity of the cell [102]. Moreover, it has been discovered that the protein serine/threonine/tyrosine kinase-1 (STYK-1) is overexpressed in SW900 NSCLC cells, which stimulates the production of Gpx4 and boosts the propagation of lung cancer cells through reducing several mitochondrial defects, ultimately leading to ferroptosis inhibition [103].

### 5.4. Ovarian Cancer

The most lethal kind of cancer in women’s reproductive systems is ovarian cancer (OVCA) [104]. Recurring, chemoresistant, and eventually fatal illnesses are the standard for most patients. The development has been related to ovarian cancer stem cells (OCSCs) [105]. OVCA incidence and resistance to treatment have been associated with CSCs. Recently, research has shown that OCSCs depend on Fe for growth and survival [106]. Ferroptosis, on the other hand, may treat cancer cells using chemicals that trigger the process such as cisplatin [96]. Previous studies have proved that the treatment of several cancer types including pancreatic, lung, colorectal, and ovarian cancers, the ferroptosis inducers such as RSL3 and RSL3A augment synergistically the anticancer effects of the drug cisplatin by blocking system Xc^−^ or Gpx4 in the body [72]. Cancer stem cells OCSCs are more vulnerable to erastin therapy than noncancer stem cells [101]. There is still much to learn about ferroptosis in OVCA, although we already recognize that docetaxel plus PARP inhibitors work synergistically with ferroptosis-inducing agents such as cyclophosphamide and vincristine in OVCA [107]. Sulfasalazine and other mature medicines have been shown to promote ferroptosis in cancer forms such as breast cancer and head and neck cancer; their therapeutic usage in OVCA is still limited because of this finding. Since these mature medications may be used for various cancers, they should be investigated for clinical use [108]. Despite continuous progress in ferroptosis research, the mechanisms underpinning the three most common protective routes still have to be enhanced and if there are more essential mechanisms are yet unknown [8].

### 5.5. Brain Tumors

There is a large concentration of peroxide-producing PUFAs in the neurological system. Ferroptosis induction was more effective in the treatment of brain tumors. In aggressive brain tumors, erastin and sorafenib may both elicit powerful cell-killing mechanisms [109]. According to a few studies, the tissue in the brain develops a defense against cell death and Nrf2 activation was also elevated [110]. Cancer cells are protected against ferroptosis development via the Nrf2–Keap1 pathway, as previously stated [111]. The Nrf2 upregulation or Keap1 depletion in glioma cells enhances oncogenic transformation. Patients who had higher levels of Nrf2 also showed a low survival rate [112]. Glioblastoma (GBM) is indeed a deadly brain tumor that has a terrible prognosis [113]. Patients die from cancer even after undergoing surgery and radiochemotherapy. Relapsed and refractory malignancies may have a connection to CSCs. GBM patients received therapy with temozolomide (TMZ) [114]. Ferroptosis was the only cell death produced by TMZ in glioblastoma stem-like cells (GSCs) throughout therapy. Ferroptosis proliferation in brain tumors was also shown by this research, which suggests that ferroptosis has a strong connection with brain tumors [115].

### 5.6. Hepatocellular Carcinoma

Hepatocellular carcinoma (HCC) cells are more susceptible to ferroptosis than normal liver cells because of the upregulation of the oncogene RAS and the higher Fe concentration compared to normal liver cells [116]. As a result, ferroptosis is thought to have a significant role in the genesis and progression of HCC. Through the latest research, it has been revealed that the influence of the p62-Keap1-Nrf2 signaling cascade on HCC is highly connected to the ferroptosis generated via elastin and sorafenib [41]. When erastin and sorafenib are used together, interfering with p62 (the substrate adaptor) increases the amount of ferroptosis that occurs in HCC cells [117]. Aside from that, erastin/anti-HCC sorafenib’s efficacy is enhanced when the Nrf2 gene is silenced [118]. Normally, in the cell’s cytoplasm, Nrf2 and Keap1 interact with one another particularly, resulting in the inhibition of Nrf2 activity [119]. The P62, when expressed in cancer cells, blocks the degradation of Nrf2 and increases its stimulation, translocation, or nucleation by inactivating Keap1, resulting in the prevention of ferroptosis in the cancer cells [120]. p53 causes ferroptosis in HCC and suppress the transcription of SLC7A11, which is a transcription factor. SLC7A11 is a rate-limiting enzyme in the production of GSH [121]. Following the inhibition of SLC7A11, the GSH synthesis is lowered and ROS generation is elevated, resulting in the induction of ferroptosis [116]. However, in cancer cells that have a p53 deficit or mutation, the connection between p53 and SLC7A11 is disrupted, resulting in increased SLC7A11 activation and ferroptosis suppression. CISD1 (CDGSH iron sulfur domain 1), also known as mitoNEET, is a protein that is anchored to the mitochondrial outer surface and is widely expressed in mitochondria-rich tissues such as the liver and heart [122]. It is involved in regulating mitochondrial Fe absorption and is found in the liver and heart. HepG2 and Hep3B human HCC cells treated with ferroptosis-inducer erastin show increased expression of the CISD1 gene compared to control cells [123]. Because of CISD1 inhibition, mitochondrial lipid peroxidation is increased and ferroptosis produced by erastin becomes more severe [1]. The pioglitazone, a Fe-sulfur cluster stabilizer that targets the CISD1 gene, reduces mitochondrial Fe uptake and OS in HCC cells [39]. CISD1 is a transcription factor that negatively controls ferroptosis by avoiding mitochondrial damage during the onset and progression of HCC [117].

### 5.7. Fibrosarcoma

The HO-1 starts heme oxidation and release carbon monoxide, Fe, and biliverdin from the heme matrix [124]. Treatment with erastin causes HO-1 to mimic the creation of internal Fe ions as well as lipid ROS, which leads to faster cell death in fibrosarcoma cells [39]. Another study revealed that in HT-1080 fibrosarcoma cells, Fe-containing porphyrin hemin accelerates ferroptotic cell death mediated by erastin in a HO-1-dependent way [125]. Moreover, the cystathionine buildup and lipid ROS formation are prevented when cysteinyl-tRNA synthetase (CARS) is knocked down, but Fe homeostasis is not disturbed [126].

## 6. Ferroptosis in Cancer Treatment

**Figure 3:**
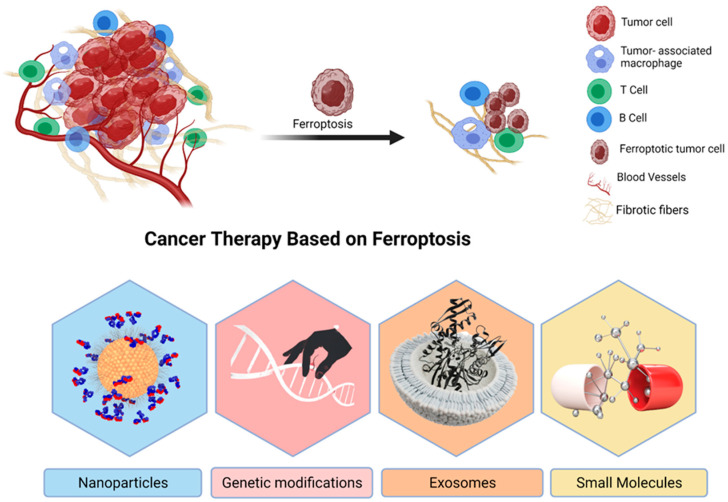
The promising use of ferroptosis for cancer treatment. A representation of ferroptosis-based cancer therapeutics, which includes small chemicals, nanoparticles, exosomes, and gene technology. It is possible to use nanomaterials as drug-inducing ferroptosis and Fe carriers in the chemotherapy combined with hyperthermia and autophagy, etc. Because exosomes are more biocompatible and less immunogenic than nanomaterials, they have a better chance of being used in clinical trials. One of the most difficult aspects of gene technology is that it may be broken down into two categories: “knockdown” and “transfection”. It will be confronted with a variety of difficulties.

Cell death is considered to be essential for maintaining homeostasis and preventing the development of over-proliferative diseases [127]. Tumor cells go through several RCDs during their growth process, including necrosis and apoptosis [41]. For cancer patients, the activation or amplification of certain kinds of RCD makes up a potentially effective and long-term approach for the therapy of their disease [43]. Most anticancer medications are now in use in clinical settings, for example, are based on typical apoptotic signaling pathways that trigger cancer cell death in cancer cells [123]. Recent research has shown that triggering ferroptosis is a helpful and potential anticancer treatment technique, and it offers new therapeutic possibilities for cancer patients [128]. Small compounds, nanomaterials, exosomes, and gene technologies based on induced ferroptosis, to name a few examples, have shown significant antitumor application potential (Figure 3).

### 6.1. Nanoparticles

In terms of precise targeting, nanoparticles (NPs) are superior since they may be used with both active and passive targeting methods. However, although nanotechnology is rapidly being employed in cancer treatment, the use of NPs-based therapy is fraught with difficulties because of factors such as inherent immunogenicity and persistent cytotoxicity [129]. There are several reasons why a combination of ferroptosis and other cancer treatments is important because of tumor complexity [130]. Ferroptosis and photothermal treatment (PTT) have been combined in a novel therapeutic method known as Sorafenib mesoporous polydopamine super-paramagnetic iron oxide NPs (SRF@MPDA-SPIO-NPs) according to recent research [131]. Ferroptosis in this system is facilitated by SPIO. SRF may also trigger cancer cell ferroptosis at the same time [132]. An adaptive PTT may also be achieved by using MPDA-NPs, which generate heat under laser emissions. For the first time, a combination of PTT and ferroptosis has been a potential anticancer therapy [126]. Ferroptosis may also be sped up by activating autophagy, which promotes ferritin breakdown and speeds up ferroptosis [123]. Nanocomposite MnO_2_@ HMCu2-xS (HMCM) was recently employed for cancer treatment using PTT-enhanced as well as autophagy-enhanced ferroptosis procedures [133]. MnO_2_ may stimulate ferroptosis in the TME by depleting GSH, and PTT can work with ferroptosis caused by HMCu2-xS to provide a synergistic effect [134]. ROS may be produced as a byproduct of ferroptosis via a ROS reaction started by the subsequent release of Mn^2+^ Which acts as an additional mechanism to ferroptosis induced by lipid hydroperoxides [135].

### 6.2. Genetic Modifications

Using gene technology in cancer treatments based on ferroptosis has been established in several studies, which may be split into two categories: gene-knockdown technology and gene-transfection technology [136]. For cancer therapeutics based on ferroptosis, it is possible that genes such as p53, Gpx4, ACSL4, Nrf2, and some others may emerge as viable targets [45].

### 6.3. Exosomes

Cancer therapies have been bolstered by nanotechnology; however, the use of nanomaterial-based cancer therapy is hampered by faults, such as residual cytotoxicity [137]. Since exosomes, which are lipid bilayer vesicles compartments measuring 30–120 nm, have excellent biocompatibility, minimal immunogenicity, and a preference for tumor migrating, they may serve as drug-delivery vectors [138]. In triple-negative breast cancer (TNBC), conventional hormone and epidermal growth factor receptor (EGFR)-targeted medication treatments are a waste of time [139]. Exosome transport and folate receptor uptake efficiency in TNBC cells have been improved by a new system termed rastin@ FA-Exo, which is engaged in the target transportation of exosomes and folate receptors [45]. Exosomes, on the other hand, may have a role in tumor cells’ resistance to ferroptosis [139]. Ferritin, a key Fe-storing protein, was found in exosomes produced from Gpxinhibitor-treated, ferroptosis-resistant cells. When ferroptosis was induced, prominin 2 levels were inversely associated with cellular Fe levels [140]. Ferroptosis is prevented in cells by constantly overexpressing a unique Fe export mechanism comprising multivascular body (MVB)/exosome transportation of ferritin and Fe from the cell. As a result, ferroptosis is prevented and intracellular Fe buildup is restricted. Ferroptosis-inducing factors are activated when this program is deactivated, either by itself or after pharmacological or genetic intervention. Modulating ferroptosis as a treatment approach in cancer has important ramifications [141]. Breast cancer cells that are exposed to proferroptosis stimuli can benefit from increased prominin-2 (a pentaspanin protein that serves as an important regulator of cell lipid dynamics) expression, which can facilitate the formation of MVBs or exosomes containing ferritin that deliver Fe from the cells [142]. It has been shown that cancer cells may resist ferroptosis initiation through the prominin-2-MVB-exosome-ferritin cascade, which may represent a novel method of ferroptosis in cancer suppression [39].

### 6.4. Small Molecules

Tumor treatment failure may be caused in part by chemoradiotherapy resistance. Ferroptosis will offer a new avenue for conventional chemotherapy and radiation to target tumor cells [143]. To treat terminal HCC, the first-line treatment is sorafenib, which induces apoptosis, inhibits proliferation, and also induces ferroptosis. Nrf2, metallothionein 1G (MT-IG), and retinoblastoma (Rb) have been shown to reduce sorafenib-induced ferroptosis [13]. Consequently, blocking these three regulators might increase sorafenib resistance [45]. xCT inhibition by SAS, a drug often used to treat arthritis and inflammatory bowel illness, results in ferroptosis, a promising new therapy for cancer [136]. The CDGSH iron sulfur domain 2 (CISD2) suppression in HNCCs may increase mitochondrial Fe^2+^ and ROS buildup, making cancer more vulnerable to SAS-induced ferroptosis [144]. To promote ferroptosis, artemisinin and its derivatives may increase the generation of ROS, regulate the Xc^−^/Gpx4 system axis, and cause ferritin breakdown in the lysosomes [145]. The activation of ferroptosis by artesunate (ART) kills PDACs and HNCCs [146]. Increasing the sensitivity of artesunate and reversing ferroptosis susceptibility in HNCCs may be achieved by inhibiting the Keap1-Nrf2 -ARE pathway [147]. As a result, ferroptosis activation is a viable method for overcoming tumor cell resistance to drugs [137]. For cancer treatment based on ferroptosis, investigational substances such as erastin and lapatinib have shown potential as therapeutic medications, as well as siramesine [40]. Ferroptosis inducers inhibited tumor growth and improved chemotherapeutic treatment sensitivity. The toxicological synergy between chemotherapy medications such as temozolomide, cisplatin, and doxorubicin and the ferroptotic inducer erastin was also observed recently. Thus, inducing ferroptosis is a unique possible cancer treatment method [148].

## 7. Conclusions

Programmed cell death seems to be a hot topic in both biological studies and medical practice at the moment. In cancer therapy, one of the most popular approaches is to target the cell death process. Ferroptosis is a newly coined term, leading to an apoptosis mechanism that exhibits distinct characteristics and has considerable promise in cancer treatment, as shown by the research. Despite the significant progress that has been achieved in recent times, there are still several issues that need to be clarified or resolved. The ferroptosis-inducing chemicals are only efficient against certain cancer cells and are ineffective against other cancer cells. It is still necessary to make efforts to categorize the malignancies that are vulnerable to ferroptosis. Having this knowledge is critical in using ferroptosis in cancer treatment. The link between ferroptosis and other cell death processes has to be defined properly under various clinical settings since it is vital to integrate diverse techniques of illness therapy to effectively cure the disease. The in vivo indicators for ferroptotic cell death are not yet available. Secondly, the implementing cell death is changed by a specific gene product is the condition for the concept of “programmed” death. Lipid peroxides are commonly regarded as the cause of ferroptotic cell death, although no one knows exactly how ferroptosis is carried out. Ferroptosis’ core trait is its Fe reliance; however, the precise involvement of Fe throughout this process remains a mystery. Cancers that are susceptible to ferroptosis are currently being classified. Cancer treatment might benefit from the use of ferroptosis. There has to be clarity in the link between ferroptosis and other cellular death processes since it is vital to integrate diverse treatment techniques for disorders.

## Figures and Tables

**Table 1:** The effects of various factors and mechanisms involved in ferroptosis.

Factors	Effects on Ferroptosis	Mechanisms Related to Ferroptosis	References
System Xc^−^	Antiferroptosis	Promotes cystine absorption	[13]
HPA5	Antiferroptosis	Prevents Gpx4 degradation	[18]
Gpx4	Antiferroptosis	Prevents lipid peroxidation reaction	[37]
MT-1	Antiferroptosis	Binds heavy metals	[38]
Ferritin	Antiferroptosis	Fe storage inside cell	[5]
IRP2	Antiferroptosis	Manages ferritin transcription	[39]
SAT1	Proferroptosis	Involved in peroxidation of arachidonic acid	[40]
ACSL4	Proferroptosis	Increases concentration of PUFA in plasma membrane	[41]
HO-1	Proferroptosis	Controls degradation of heme and release of Fe	[42]
p53	Proferroptosis	Tumor suppressor and expression of SLC7A11 gene	[43]
NOX	Proferroptosis	Promotes ROS production	[39]
CARS	Proferroptosis	Protein translation via charging of tRNAs with cystine	[44]
TfR1	Proferroptosis	Promotes the Fe uptake	[45]

## Abbreviations

**Full Form**	**Abbreviation**
Acyl-CoA synthase family member 2	ACSF2
Acyl-CoA synthetase long-chain family member 4	ACSL4
Apoptosis-inducing factor	AIF
Apoptosis-inducing factor mitochondria associated 2	AIFM2
Apoptosis-inducing factor-1	AIF-1
Arachidonate 15-lipoxygenase	15-LOXs
Arachidonoyl peroxidation	AA
Artesunate	ART
Autophagy related-5, autophagy related-7, and nuclear receptor coactivator autophagy related-4 pathways	ATG5-ATG7-NCOA4
Autophagy-dependent ferroptosis	ADF
Autophagy-related protein-13	ATG-13
Autophagy-related-3 protein	ATG-3
BH3 interacting domain death agonist	BID
BRCA1-associated protein-1	BAP-1
Carbonic anhydrase-9	CA9
CDGSH iron sulphur domain 2	CISD2
CDGSH iron sulphur domain 1	CISD1
Clear-cell carcinoma	CCC
Colorectal cancer	CRC
Competitor endogenous RNAs	ceRNAs
Cysteinyl-tRNA synthetase	CARS
Diffuse large B-cell lym-phoma	DLBCL
Dipeptidyl peptidase-4	DPP4
Divalent metal transporter-1	DMT1
Electron transport chain	ETC
Ferritin heavy chain-1	FTH-1
Ferritin light chain	FTL
Ferroportin	Fpn
Ferroptosis suppressor protein-1	FSP-1
Gamma-glutamyl-cysteine	g-GCS
Glioblastoma steam-like cells	GSCs
Glutaminase-2	GLS2
Glutathione	GSH
Glutathione peroxidase 4	Gpx4
Head and neck squamous cell carcinomas	HNSCC
Heat shock protein beta-1	HSPB1
Heat shock protein-5	HSPA5
Hepatocellular carci-noma	HCC
Hepatocyte growth factor-2	HGF-2
Hypoxia-inducible factor	HIF
Hypoxia-inducible factor-1	HIF-1
Hypoxia-inducible protein-2	HIG-2
Iron	Fe
Iron regulatory proteins	IRP1
Iron response element-binding protein-2	IREB-2
Kaplan–Meier	KM
Labile iron pool	LIP
Lung cancer	LC
Lysophosphatidylcholine acyltransferase 3	LPCAT3
Metallothionein 1G	MT-IG
Mitochondrial ferritin	FTMT
Mucin 1 cell surface associated	MUC1-C
Multiple myeloma	MM
NADPH oxidase 1	NOX1
Nanoparticles	NPs
Nicotinamide adenine dinucleotide diphosphate oxidase-1	NADPH Oxidase/NOX-1
Noncoding RNA	ncRNA
Nuclear receptor coactivator-4	NCOA-4
Ovarian cancer	OVCA
Ovarian cancer stem cells	OCSCs
Oxidative stress	OS
P62-Kelch-like ECH associated protein 1- nuclear factor erythroid 2-related factor 2	p62-Keap1-Nrf2
Pancreatic cancer	PC
Pancreatic ductal adenocarcinoma	PDACs
Pentaspanin protein	Prominin-2
Photo-thermal treatment	PTT
Polyunsaturated fatty acids	PUFAs
Prostaglandin-endoperoxide synthase-2	PTGS-2
Radiation-induced bystander effect	RIBE
RAS selective lethal	RSL3
Receptor-interacting protein kinase-1	RIPK-1
Regulated cell death	RCD
Renal cell carcinoma	RCC
Retinoblastoma	Rb
Serine/threonine/tyrosine kinase-1	STYK-1
Signal transducer and activator of transcription 3	STAT3
Six transmembrane epithelial antigens of the prostate-3	STEAP-3
Solute carrier family 11 member-A2	SLC11A2
Solute carrier family 7 member 11	SLC7A11
Spermidine/spermine N1-acetyltransferase 1	SAT1
Squamous cell carcinomas	SCCs
Temozolomide	TMZ
TF receptor-1	TFR-1
The activating transcription factor-4	ATF4
Transferrin	TF
Transferrin receptor	TFRC
Transferrin receptor-1	TFR1
Tricarboxylic acid	TCA
Tumor microenvironment	TME
Tumor-associated macrophages	TAMs
Tumor-cell-released microparticles	RT-MPs
Ubiquinone	CoQ10
Voltage-dependent anions channels	VDACs
Zinc–Iron regulatory protein family 8/14	ZIP8/14
